# Editorial: Predictors for aggressiveness of papillary thyroid carcinoma

**DOI:** 10.3389/fendo.2024.1363836

**Published:** 2024-01-18

**Authors:** Emese Mezosi

**Affiliations:** I^st^ Department of Internal Medicine, Clinical Center, University Medical School of Pecs, Pecs, Hungary

**Keywords:** papillary thyroid cancer (PTC), aggressiveness, lymph node metastasis, predictive model, extrathyroidal extension (ETE)

Papillary thyroid cancer is the most common endocrine malignancy with a good prognosis in most cases. The increasing incidence of papillary thyroid cancer worldwide underlies the importance of early selection of those patients who have aggressive tumors and require more extended surgery and/or postoperative therapy. The preoperative risk stratification is not optimal due to the limitations of imaging methods and lack of information about the histological parameters and molecular background. The establishment of effective predictive models may help the decision-making in individual cases.

Feng et al. developed machine-learning models for predicting occult central lymph node metastasis (CLNM) based on clinicopathological and sonographic characteristics. The nine top-rank variables were tumor size, margin, extrathyroidal extension, gender, echogenic foci, shape, number, lateral lymph node metastasis, and chronic lymphocytic thyroiditis. Comparing eight machine learning models for the prediction of CLNM, the best performance was achieved by the Random Forest model. Prophylactic central lymph node dissection is recommended in high-risk patients.

Another paper in this Research Topic also dealt with the preoperative prediction of CLNM. They have combined conventional and contrast-enhanced ultrasound and developed and validated a web-based dynamic nomogram to stratify high-risk and low-risk groups which may help to refine surgical approach (Chen et al.).

Li et al. proposed a similar study to predict the risk of cervical lymph node metastasis in multifocal papillary microcarcinomas. Age, gender, the size of the tumor, and ultrasound characteristics were used to create a model that demonstrated a significant clinical benefit.

Zhang et al. further investigated the possible selection of high-risk patients with papillary microcarcinomas in a large dataset. Active surveillance has been accepted in a subgroup of PTMC (1). In their nomogram model, male sex younger age, larger tumor diameter, bilaterality, and multifocality were independent predictors of the high-risk group where surgery is recommended.

According to the current guideline, prophylactic central neck dissection is not routinely recommended for small (T1 or T2), noninvasive, cN0 PTC (1), however, the rate of CLNM was 36.4%~64.7% in clinically node-negative PTC. The decision about the type of surgery may be challenging in this group of patients. Wang et al. conducted a large retrospective study to identify intermediate-risk patients with more than 5 metastatic central lymph nodes in T1-2 N0 PTC. They have found that if more than two metastatic prelaryngeal and/or pretracheal lymph nodes occurred, 71.2% of patients belonged to the intermediate-risk group where total thyroidectomy and ipsilateral CLN dissection should be performed.

Metastasis in lymph nodes posterior to the right recurrent laryngeal nerve (LN-prRLN) is a crucial component of the CLNMs, however, surgical removal is difficult, the complication rate is high, and the preoperative assessment is imperfect. The prediction of these CLMNs would be especially helpful for surgeons. Gong et al. investigated the possible prediction of this special localization CLNM in their retrospective study and demonstrated that patients with metastasis in LN-prRLN were younger with larger tumor size, CEUS centripetal perfusion pattern, the presence of CLNM detected by ultrasound and metastasis in LN anterior to the right recurrent laryngeal nerve were independent risk factors; complete CLN dissection is recommended in these cases.

The number of metastatic lymph nodes is influenced by the extent of LN dissection. Lymph node ratio (LNR) means the number of metastatic LNs divided by the total number of removed lymph nodes during surgery. This ratio has been used to estimate prognoses in several solid tumors. Ma et al. investigated the diagnostic efficacy of LNR to predict the risk of recurrence in N1a PTC patients. The optimal LNR cutoff values for structural and biochemical recurrence were 0.75 and 0.80, respectively. The authors recommended the evaluation of lateral neck lymphadenopathy and RAI treatment in those with LNR≥0.75.

The prediction of central and lateral neck lymph node involvement was the topic of a subsequent paper in PTC patients with thyroid capsular invasion (TCI) which includes both microscopic and macroscopic extrathyroidal extension. Patients with TCI were older and had larger tumor sizes, and the presence of bilateral disease and multifocality was more common. Interestingly, Hashimoto thyroiditis was more common in the no-TCI group. The occurrence of CLNM was significantly higher in the TCI than in the no-TCI group (77.1% vs. 34.2%). The involvement of lateral lymph nodes was evaluated in patients with CLNM and was much higher in patients with TCI, 29.3% vs. 16.4%. Nomogram models were constructed for the prediction of CLNM and LLNM in TCI and no-TCI patients to aid clinical decision-making in the management of PTC patients (Yang et al.).

The extrathyroidal extension (ETE) of the tumor as an important prognostic factor was the subject of investigation in the paper of Xu et al. who retrospectively evaluated an extremely large patient population, more the 100,000 PTC patients from the National Cancer Institute’s SEER database. The role of ETE is a controversial issue; based on its limited impact on prognosis, minimal ETE has been removed from the staging system in the eighth edition of the AJCC. ETE was categorized into none, capsule, strap muscles, soft tissue, and other organs. ETE into soft tissues or other organs proved to be a bad prognostic factor for both overall survival and thyroid cancer-specific survival. Invasion into the strap muscles also impaired the overall survival of patients with age ≥55 years or >2 cm tumor size which should be taken into consideration in the treatment plans.

The work of Wang et al. is thematically different from the previous ones, the authors evaluated the diagnostic role of NDRG family member 3 as a serum tumor marker in patients with PTC, nodular goiter, and control subjects. NDRG3 is a downstream regulator of MYC which is a human proto-oncogene, involved in the development and invasion of many tumors, including PTC. The serum level of NDRG3 was lower in PTC patients, especially in patients with lymph node metastases and/or extrathyroidal extension. To evaluate the clinical relevance of these findings needs further investigations.

The 10 articles in this Research Topic contributed to our knowledge regarding prognostic factors in PTC. The more general use of artificial intelligence and machine learning provides a new option to proceed in patient management and implement personalized medicine.

## Author contributions

EM: Writing – original draft, Writing – review & editing.
